# Stability and flexibility of odor representations in the mouse olfactory bulb

**DOI:** 10.3389/fncir.2023.1157259

**Published:** 2023-04-20

**Authors:** Haran Shani-Narkiss, David Beniaguev, Idan Segev, Adi Mizrahi

**Affiliations:** ^1^The Edmond and Lily Safra Center for Brain Sciences, The Hebrew University of Jerusalem, Jerusalem, Israel; ^2^Department of Neurobiology, The Hebrew University of Jerusalem, Jerusalem, Israel

**Keywords:** odor representations, mitral cells, two-photon imaging, mice, plasticity, anesthesia, stability

## Abstract

Dynamic changes in sensory representations have been basic tenants of studies in neural coding and plasticity. In olfaction, relatively little is known about the dynamic range of changes in odor representations under different brain states and over time. Here, we used time-lapse *in vivo* two-photon calcium imaging to describe changes in odor representation by mitral cells, the output neurons of the mouse olfactory bulb. Using anesthetics as a gross manipulation to switch between different brain states (wakefulness and under anesthesia), we found that odor representations by mitral cells undergo significant re-shaping across states but not over time within state. Odor representations were well balanced across the population in the awake state yet highly diverse under anesthesia. To evaluate differences in odor representation across states, we used linear classifiers to decode odor identity in one state based on training data from the other state. Decoding across states resulted in nearly chance-level accuracy. In contrast, repeating the same procedure for data recorded within the same state but in different time points, showed that time had a rather minor impact on odor representations. Relative to the differences across states, odor representations remained stable over months. Thus, single mitral cells can change dynamically across states but maintain robust representations across months. These findings have implications for sensory coding and plasticity in the mammalian brain.

## Introduction

Mitral cells (MCs) are the main projection cells of the olfactory bulb (OB). The representations of odors by MCs are a result of bottom-up input from olfactory sensory neurons in the epithelium, top-down projections from multiple higher brain areas and local computations carried out by the rich inhibition in the OB (Urban and Sakmann, 2002; Nunez-Parra et al., 2013; Gschwend et al., 2015; Economo et al., 2016; Reshef et al., 2017; Vinograd et al., 2017a). Much of what we know about MC coding comes from experiments conducted in different states of the animal - anesthetized, awake, passively or actively sniffing. The extent to which different states represent similar or mutually exclusive information remains an open question (Rinberg et al., 2006; Davison and Katz, 2007; Kato et al., 2012; Blauvelt et al., 2013; Kollo et al., 2014).

The dramatic transition of neural representations from the awake to the anesthetized state received limited attention in olfaction. While several findings described this transition at the single cell level (Rinberg et al., 2006; Davison and Katz, 2007; Blauvelt et al., 2013; Kollo et al., 2014), only few also described how the transition between anesthetized and awake states changes population activity as a whole (Kato et al., 2012; Bolding et al., 2020). For example, Kato et al. (2012) used calcium imaging and described a monotonic, dis-inhibitory, effect of anesthesia on MCs activity. However, and somewhat anecdotally, over the years we have observed that the transition between anesthesia and wakefulness is not monotonic but rather quite variable (Vinograd et al., 2017a; Shani-Narkiss et al., 2020; Kudryavitskaya et al., 2021). Therefore, we set out to re-evaluate the effects of anesthesia versus wakefulness on odor responses by MCs. We used a more sensitive calcium probe [GCamp6f (Chen et al., 2013)] than the one used in the past (i.e., GCamp3), and a larger odor set that includes both simple and complex odors. We conducted time-lapse experiments, and imaged activity from the same MCs weeks apart in both anesthetized and awake states. Our results show that the transition between states is a complex interplay of extensive changes that leads to qualitatively different odor representations by MCs between anesthetized and awake states. Yet, the distinct representations of odors in either anesthetized or awake states remained similar across long periods of time, demonstrating the stability of odor codes when measured within a specific state. Together, our work demonstrates the breadth of flexibility and stability of odor representations by MCs.

## Results

### Odor representations in awake mice are balanced across the population

**FIGURE 1:**
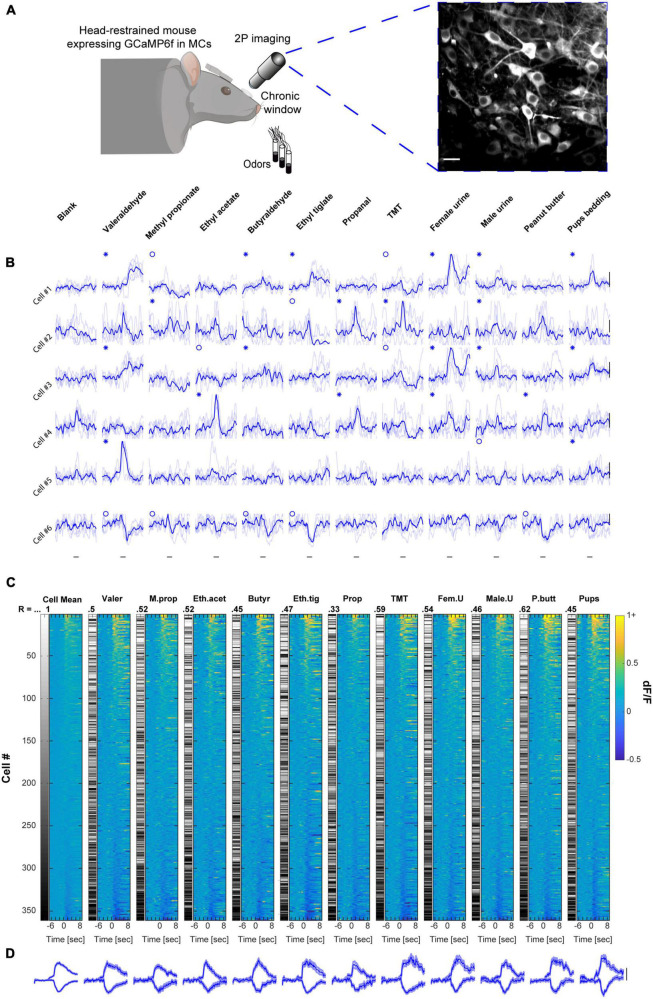
Mitral cell (MC) responses in the awake state: balanced population response profile across odors. **(A)** Schematic illustration of the setup and a 2-photon micrograph of a representative field of MCs expressing GCamp6f. Scale bar = 25 μm. Created with BioRender.com. **(B)** Examples of odor-evoked calcium transients in an awake mouse, from 6 MCs in response to 11 odors. Odor stimulation is denoted as a black horizontal line below all traces (2 s). Thin traces are five single trials; thick traces are means. Blue asterisks denote significant excited responses and blue circles denote significant suppressed response. Scale – vertical black lines, 30% dF/F. **(C)** Color coded columns shows all the data recorded from awake mice, sorted by magnitude per each stimulus separately. Greyscale columns - Ranks of Cells’ average response fitted to each cell-odor pair. Spearman correlation coefficients denote the rank correlation between the MC population average response and the population response to each one of the odors. Full odor names are depicted in panel **(B)**. Left color column (“Cell Mean”) depicts the mean response magnitude of each cell for the different odors. The left greyscale column, serves as a scale bar of cell identity. **(D)** Average traces of population S and E responses for each of the odors presented. Scale – vertical black line, 20% dF/F. Traces are presented as mean response (thick lines), and shadows are the SEM. Each trace represents 7 s pre-stimulus, 2 s stimulus presentation, and 7 s post-stimulus.

To evaluate odor representations, we used *in vivo* two-photon calcium imaging of awake, head-restrained, mice expressing GCamp6f in MCs (Figure 1A, *N* = 11 mice, *n* = 361 MCs). We presented mice with a set of 11 odorants, composed of 6 monomolecular odors (Valeraldehyde, Methyl propionate, Ethyl acetate, Butyraldehyde, Ethyl tiglate, and Propanal), and 5 biologically relevant odors (TMT, Female urine, Male urine, Peanut butter, and Pups bedding; Figure 1B). The use of GCamp6f allowed us to measure both excited as well as suppressed calcium transients in MCs (hereafter referred to as “E” or “S” responses, respectively), both of which were abundant in our dataset. Odor responses by individual MCs were highly heterogeneous but well balanced across the population of MCs for this panel of 11 odors. Single MCs rarely showed high selectivity to one specific odor (Figure 1B), and different odors elicited surprisingly similar levels of activity in the MCs population as a whole (Figures 1C, D). To evaluate this population similarity, we first calculated the mean response for each cell by taking the average dF/F value across all odor responses. We then sorted all cells by their mean response magnitude (Figure 1C, “Cell Mean,” left column), and compared this mean population response to the population response of each odor alone using Spearman correlation (Figure 1C; 11 odor matrices; R values and bar codes next to each matrix show the cell’s identity based on its rank in the mean response). The average correlation between the mean population response and the response of the population for each odor was 0.5 ± 0.078 (mean ± SD). The relatively low correlation values suggest a balanced “division-of-labor” by the population of MCs as a whole across this panel of odors, as different cells participate in the coding of different stimuli rather than the same group of cells dominating the representation of all stimuli. Furthermore, the low variance of the distribution of correlation values (i.e., coefficient of variation = 0.156) suggests equal representation by the population of MCs across stimuli (see also similarity between population averaged traces in Figure 1D).

### Odor representations in anesthetized mice are not balanced across the population

**FIGURE 2:**
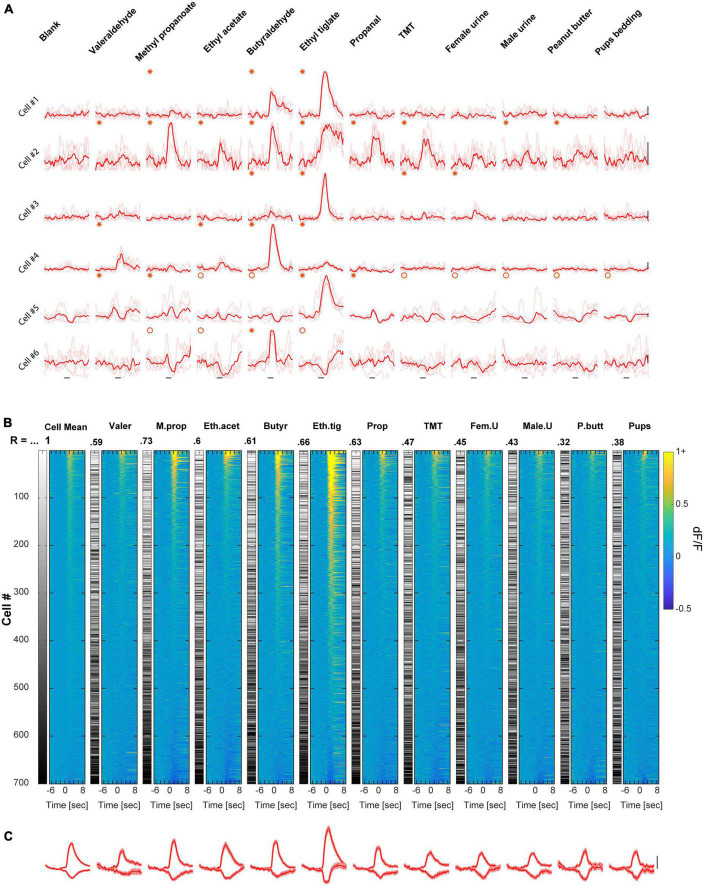
Mitral cell (MC) responses in the anesthetized state: unbalanced response profile across odors. **(A)** Examples of odor-evoked calcium transients from an anesthetized mouse, for 6 neurons and 11 odors. Odor stimulation is denoted as a black horizontal line below all traces (2 s). Thin traces are five single trials; thick traces are means. Red asterisks denote significant excited responses and red circles denote significant suppressed response. Scale – vertical black lines, 30% dF/F. **(B)** Colored columns- All data recorded from anesthetized mice, sorted by magnitude per each stimulus separately. Greyscale columns- Ranks of Cells’ average response fitted to each cell-odor pair. Spearman correlation coefficients denote the rank correlation between the MC population average response and the population response to each one of the odors. Full odor names are depicted in panel **(A)**. **(C)** Average traces of population S and E responses for each of the odors presented. Scale – vertical black line, 20% dF/F. Traces are presented as mean response (thick lines), and shadows are the SEM. Each trace represents 7 s pre-stimulus, 2 s stimulus presentation, and 7 s post-stimulus.

To evaluate odor representations under anesthesia, we imaged MCs responses to the same odor set as in the awake state in a separate group of mice using ketamine/domitor anesthesia (see section “Materials and methods”) and performed a similar analysis to the one described above (*N* = 20 mice, *n* = 700 MCs). In anesthetized mice, E responses were far more abundant than S responses, and MCs population responses were no longer balanced across odors. Some odors elicited large magnitude calcium transients (Figure 2A - ethyl tiglate), whereas other odors elicited weak responses (Figure 2A - valeraldehyde 2A). This heterogeneity was also evident at the response profile of the population, where the extent of population activity differed considerably between different odors (Figures 2B, C). The large differences in odor representations are also evident as higher variation of the Spearman correlation values as compared to wakefulness (Figure 2B, R values, mean ± SD = 0.53 ± 0.13; coefficient of variation = 0.24).

### The transition between wakefulness and anesthesia reshapes odor representations – Comparing different mice, different neurons, in different states

**FIGURE 3:**
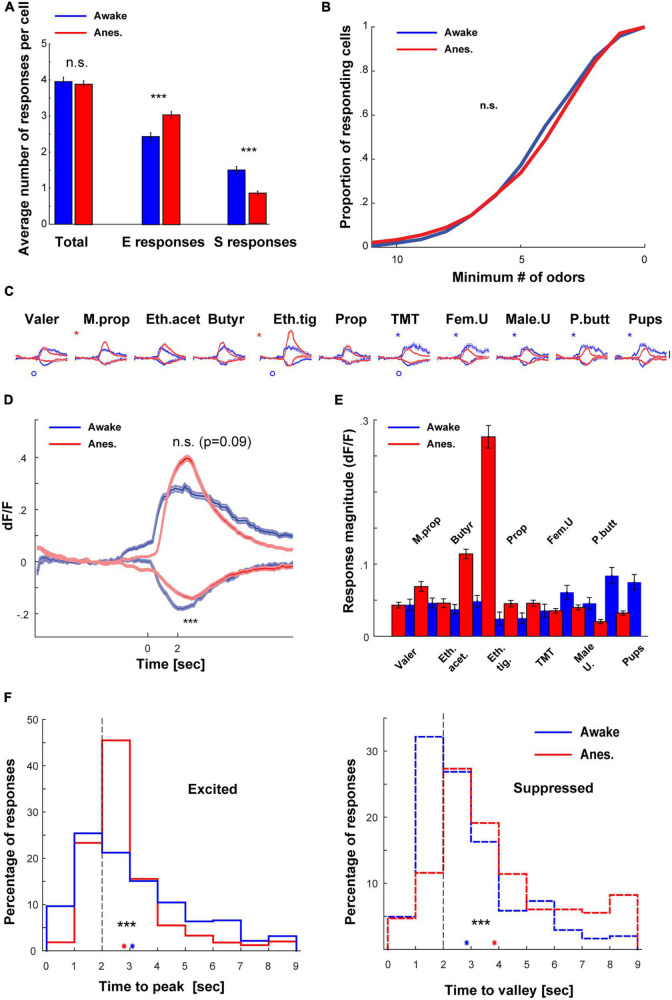
Comparison of the awake vs. anesthetized datasets. **(A)** Bar graphs summarizing the differences in number of responses per cell between awake and anesthetized mice. **(B)** Cumulative distribution of the proportion of mitral cells (MCs) responding to 0–11 odors, in wakefulness (blue) and anesthesia (red). MCs responsiveness profile was not different between awake and anesthetized mice (*p* = 0.063, Two-sample Kolmogorov–Smirnov test). **(C)** Average traces of S and E responses per each one of the odors presented to awake (blue) and anesthetized (red) mice (mean ± SEM). Scale – vertical black line, 20% dF/F. Asterisks denote differences in the E responses and circles in S responses. **(D)** Traces of S and E responses averaged across all odors presented to awake and anesthetized mice (mean ± SEM). **(E)** Mean dF/F values, averaged throughout the response window over all cells included in the datasets of both awake and anesthetized mice. **(F)** Left- histograms depicting times to reach maximum value for E responses in wakefulness (*n* = 882 responses, *N* = 11 mice) and under anesthesia (*n* = 2130, *N* = 20 mice) mice. Responses in anesthetized mice peaked significantly earlier. Right- histograms depicting times to reach minimum value for S responses in awake (*n* = 547 responses) and anesthetized (*n* = 596 responses) mice. Responses in awake mice reached minimum value significantly earlier. Blue and red asterisks denote the mean time to extremum for responses recorded in awake and anesthetized mice, respectively.

To evaluate the transition between wakefulness and anesthesia we compared between the two separate datasets mentioned above, but now focusing only on responsive cell-odor pairs (all the significant responses are plotted in Supplementary Figure 1A). Under anesthesia, there were generally more E responses and less S responses as compared to wakefulness (Figure 3A, for specific odor dependencies see Supplementary Figures 2B, C). However, these effects canceled each other such that the total responsiveness was not significantly different between states (Figure 3A- “total”; Figure 3B). Differences in response magnitudes between the states also changed across the odor set, as evident from the average calcium traces of all neurons (Figure 3C; see also Figures 1D, 2C). The trial to trial variability was higher in the awake state as compared to anesthetized state (Supplementary Figure 1B). Averaging the total E and S responses by all neurons to all odors shows that the magnitude of E responses was slightly higher (albeit not significantly) under anesthesia, and the average magnitude of S responses was weaker (Figure 3D; magnitude integrated across a 4 s response-window). The net population response magnitudes (i.e., average over the whole dataset regardless of the type or significance of response), were generally lower and more balanced across odors in the awake state as compared to the anesthetized state (Figure 3E). This balance is also expressed as lower coefficient of variation in the awake state (Mean ± STD, Awake: 0.048 ± 0.019; Anesthetized: 0.070 ± 0.073; Coefficient of variance, Awake: 0.39; and Anesthetized: 1.04).

We also analyzed the similarity of representation among neurons by calculating the signal correlation between odor response vectors of all possible neuronal pairs. We calculated pairs of cells that were imaged from the same animal, which were imaged in the two states (see section “Materials and methods”). The mean signal correlation between all pairs of neurons in the awake state were low (0.0884 ± SEM, 4889 pairs). These rather low signal correlation values were significantly higher in the anesthetized state (0.4122 ± SEM, 4889 pairs). This data shows that individual neurons’ tuning properties are more similar during anesthesia. Finally, odor responses across states differed in their temporal dynamics. Specifically, under anesthesia, E responses peaked earlier while S responses peaked later (Figure 3F). Such reshaping of activity during wakefulness suggests a normalization-like computation of odor representation by MCs. Notably, these results were unexpected based on previous reports (Kato et al., 2012) (see section “Discussion”).

### The transition between wakefulness and anesthesia reshapes odor representations – Comparing the same neurons in different states

**FIGURE 4:**
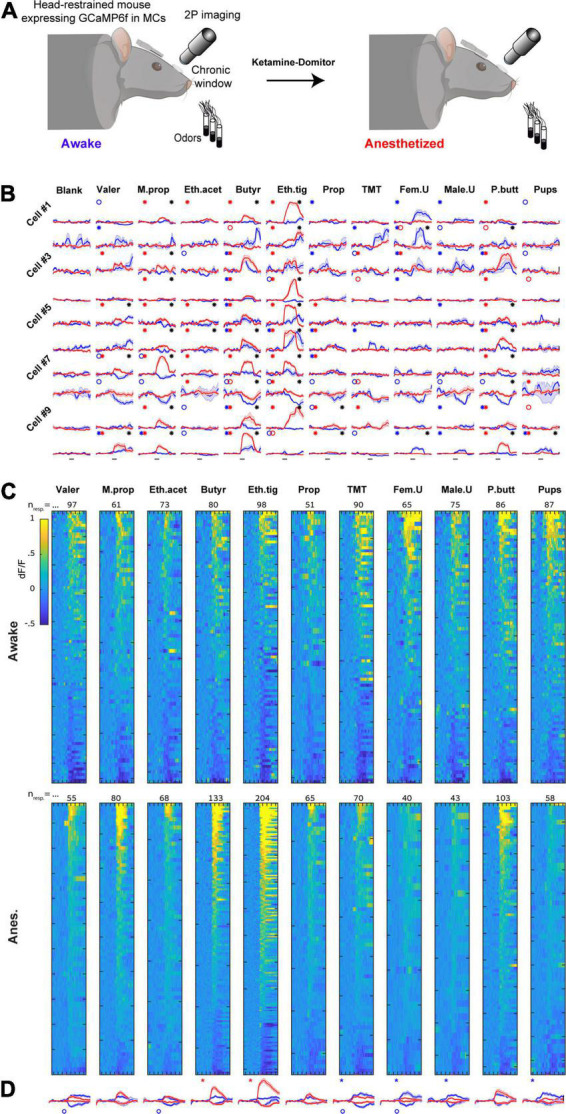
Imaging the same mitral cells (MCs) in awake and anesthetized states. **(A)** Schematic illustration of the experiment conducted to follow the same MCs transitioning from awake to the anesthetized state. Created with BioRender.com. **(B)** Examples of odor-evoked calcium transients before (blue) and after (red) anesthesia from 10 neurons in response to 11 odors. Odor stimulation is denoted as a black horizontal line bellow all traces (2 s). Traces are mean + - SEM calculated over five single trials for each time point. Blue/red asterisks denote significant E responses and blue/red circles denote significant S response in awake or anesthetized sessions, respectively. Black asterisks mark a statistically significant difference between conditions. Scale – vertical black lines, 30% dF/F. **(C)** Significant odor responses of all neurons, sorted by response magnitude. Responses were recorded from the same MCs in either awake (top) or anesthetized (bottom) sessions. The total number of responses per stimulus is depicted above each column. Odor presentation (2 s) is marked by a red line at the bottom of each column. **(D)** Average traces of S and E responses for each one of the odors presented in awake (blue) and anesthetized (red) sessions (mean + - SEM). Responses are depicted in a paired manner (see section “Materials and methods”). Scale – vertical black line, 20% dF/F. Each trace represents 7 s pre-stimulus, 2 s stimulus presentation, and 7 s post-stimulus.

To characterize more accurately the differences in odor responses between states, and in order to follow the actual transition neurons undergo, we next imaged odor evoked activity from the exact same MCs in both awake and anesthetized states and analyzed the data in a paired fashion (Figure 4A). Responses of 10 representative MCs from one mouse are shown in Figure 4B and all significant responses from all mice in both states are shown in Figures 4C, D (the full dataset is composed of 2739 cell-odor pairs from *n* = 249 MCs, *N* = 7 mice).

**FIGURE 5:**
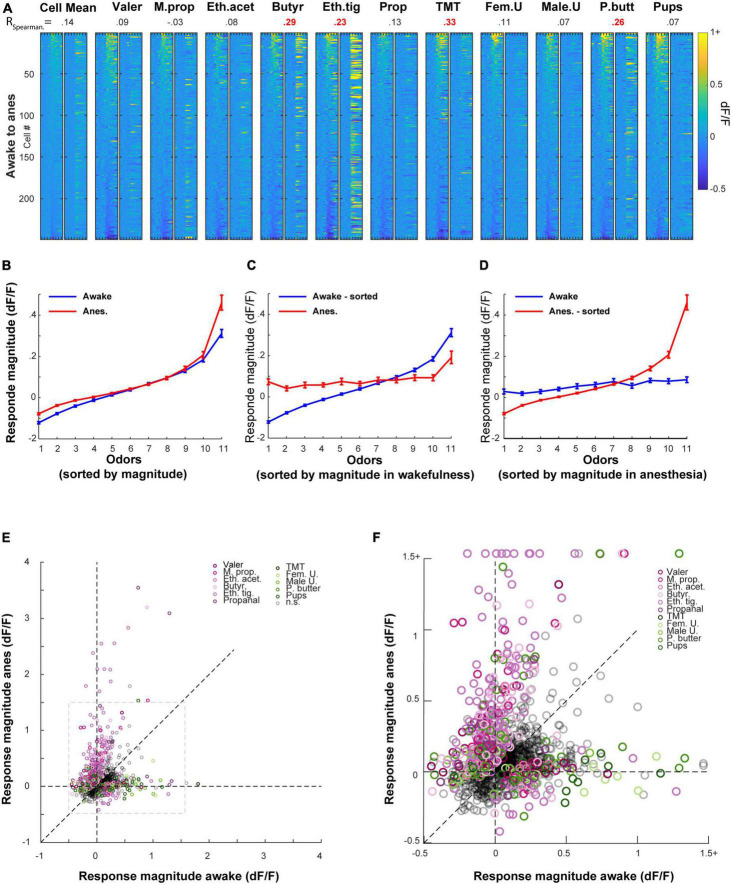
Odor responses in the awake state are poor predictors of the response in the anesthetized state, and *vice versa*. **(A)** All cell odor pairs recorded in both awake and anesthetized sessions, sorted by the response magnitude measured in the awake session. For each odor, the left column shows the sorted cell-odor pairs in the awake session and the adjacent right column shows the same cell-odor pair under anesthesia. Odor presentation (2 s) is marked by a red line at the bottom of each column. Correlation values denote the Spearman correlation between the cell-odor pairs in both states, where statistically significant correlations are in red. **(B)** Response magnitude in ranked order, before and after anesthesia. Cell odor pairs were sorted separately for awake and anesthetized recordings. **(C)** Response magnitude in ranked order, before and after anesthesia. Cell odor pairs were sorted in a paired manner according to their response in the awake recording session. **(D)** Same as C, but pairs were sorted in a paired manner according to their response in the anesthetized recording session. **(E)** Response magnitudes of all responsive cell-odor pairs before and after anesthesia. Color dots mark odor identity of responses that differed significantly between states (∼40% of all significant responses in either state). Gray dashed square marks the area that is magnified in panel **(F)**. **(F)** Magnification of the dashed gray square in panel **(E)**.

First, we repeated the same analyses that we performed in separate mice (i.e., unpaired data) on the data imaged in the same mice (i.e., paired data). We found similar effects in the paired data as compared to those we found in the unpaired data (Supplementary Figure 3, compare to Figure 3). Second, we examined how individual MCs changed their response profile between states. To do so, we sorted all cells according to their rank in one state, and plotted the exact same cell-odor pairs’ response in the other state (Figure 5A and Supplementary Figure 4A). The rank of a cell in one state (determined by its average response magnitude to all odors) had almost no indication on its rank in the other state (Figure 5A; Left column). This change between the states was evident across all odors (Figure 5A; expressed as low Spearman correlation values; shuffling odor and cell identity collapsed all correlations to 0).

Third, to characterize the tuning curves of MCs across states, we sorted odor responses according to their magnitude in each state separately, regardless of odor identity (Figure 5B). This sorting shows that the range of responses in the anesthetized state was both larger and shifted upward as whole (Figure 5B). This result is consistent with the changes in magnitudes we reported above (i.e., Supplementary Figures 3A–C). Fourth, to evaluate whether the tuning curve of single neurons is preserved between states or not, we sorted all cell-odor pairs in one state, and paired their cognate response in the other state (Figures 5C, D). In this configuration, the tuning curve of the unsorted state became nearly flat, indicating that odor responses in one state bear little similarity to the responses in the other state (Figures 5C, D).

Finally, to evaluate how MCs preserve their molecular receptive range (i.e., the responses to specific odors) following the transition between states, we plotted all cell-odor pair responses in the awake state vs. their responses in the anesthetized state. Once again, we have found that response magnitude in one state carried little information regarding response magnitude in the other state (Figures 5E, F; note the small number of dots along the diagonal). Interestingly, response magnitudes were generally larger for natural odors in the awake state (Figures 5E, F- green hues), yet larger for artificial odors in the anesthetized state (Figure 5E- purple hues; Supplementary Figure 3). In summary, our paired analyses suggest that the transition between states caused considerable changes in the tuning curves and specific molecular receptive range of MCs. Since this reshaping of representations was substantial, we were prompted to ask about the extent to which activity in one state carries information about the other state.

### Odor representations in one state are poor predictors of representations in the other state

We next tested the information carried by the population responses in either state as measured by the ability of a downstream decoder to predict odor identity in each state. Previous reports suggested that coding efficiency is better in the awake state, where activity is sparser (Kato et al., 2012). Thus, we expected that during wakefulness, MCs coding will be more informative about odor identity as compared to the anesthetized state. Our results showed the contrary.

**FIGURE 6:**
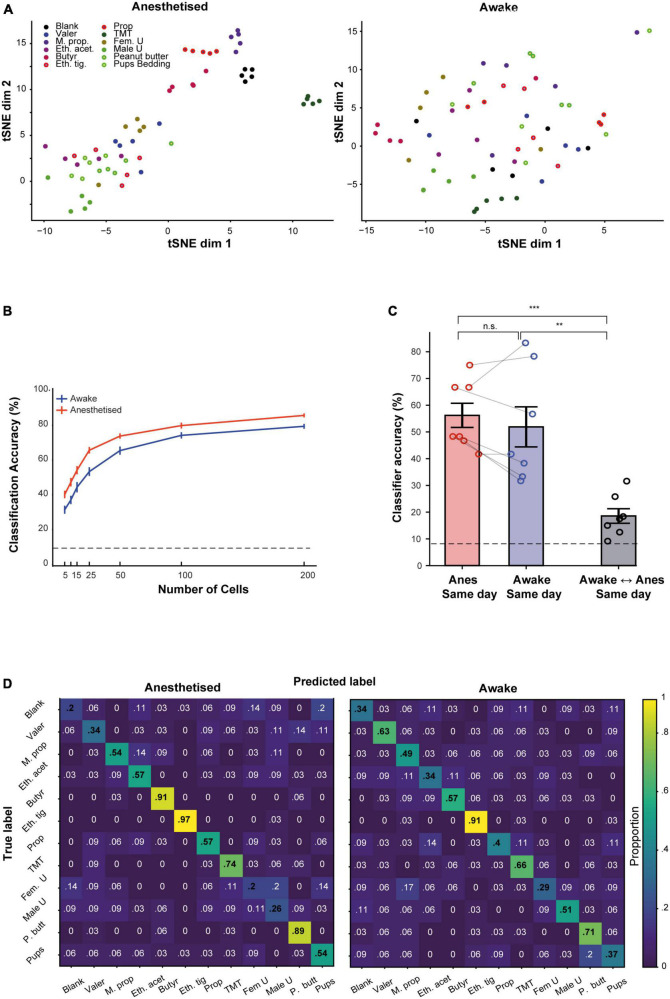
Odor representation by the population of mitral cells (MCs) in awake and anesthetized states. **(A)** Two-dimensional t-SNE representation of neural responses. Data represent the pooled MC responses in one example mouse, during anesthesia (left) and wakefulness (right). Each dot is a trial; each color is an odor. **(B)** The accuracy of a linear classifier in identifying the correct odor, as a function of the number of MCs used for the task. MCs are randomly pooled from all mice. Error bars are SEMs calculated over 40 simulations. Dashed line represents chance level. **(C)** The average accuracy of a linear classifier for different training-testing configurations, using data collected within the same day. **(D)** Confusion matrices for a classifier that was trained to distinguish between different odors, averaged over all seven mice in anesthetized (left) and awake (right) sessions. **p < 0.01; ***p < 0.001.

We initially visualized our population data using t-SNE. The pattern of clustering in the t-SNE plots suggested that responses were seemingly more separated in the anesthetized as compared to the awake state. Figure 6A shows t-SNE plot from one population of neurons in both states, calculated separately for each state. To quantify these differences, we first trained a linear classifier using MCs recorded from all the mice in our data, pooled together and randomly drawn in different group size (using different number of MCs per group). We then assessed the classifier’s ability to correctly classify the odor identity based on the population responses within each state. The average classifiers performance was slightly higher in the anesthetized state as compared to the awake state for all group sizes we assessed (Figure 6B). To further quantify these results, we trained a classifier for each mouse separately in several different configurations (Figure 6C; the detailed odor classification confusion matrices of the average of all mice is shown in Figure 6D). The highest accuracy (56%) was achieved for classification in anesthetized animals, but the average classification for the awake sessions was only slightly lower (52%), and not significantly different. However, the average classification across states collapsed to 18% and was significantly lower than the two other configurations. This result demonstrates that the representation of odor information by MCs between wakefulness and anesthesia is qualitatively different.

### Odor representations by MCs are stable over time

**FIGURE 7:**
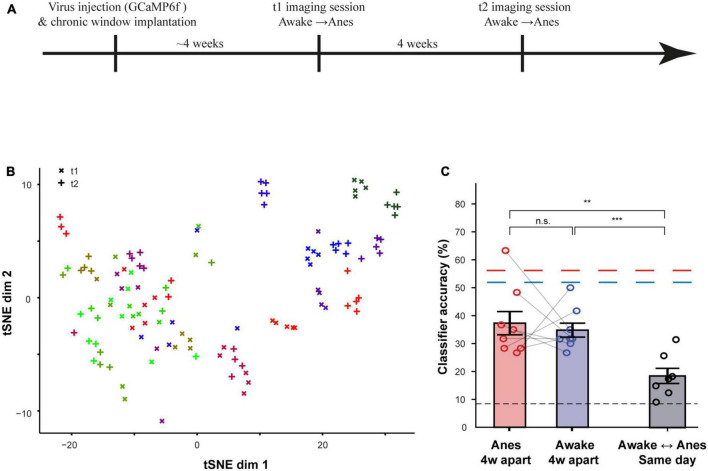
Odor representations are stable over time. **(A)** Time-line for the experiment used to assess stability and flexibility of odor representations in both awake and anesthetized states, 4 weeks apart. **(B)** Odor representation as t-SNE plots across the experiment from one example mouse in two anesthetized recording sessions, 4 weeks apart. **(C)** The average accuracy of a linear classifier for different training-testing configurations, using data collected in sessions that were 4 weeks apart. Results from the classifier using data collected within the same day are plotted as dashed lines for comparison. Black Dashed line represents chance level. **p < 0.01; ***p < 0.001.

A second aim of this work was to assess how preserved are odor representations over time. To do so, we imaged the same MCs 4 weeks apart, in both the anesthetized and awake states (Figure 7A; *N* = 4 mice; *n* = 126 MCs). An example from one anesthetized mouse is shown as a t-SNE visualization in Figure 7B. This visualization suggested that responses are well-preserved over 4 weeks, albeit with some small representational drift (Figure 7B, compare same color labels of ‘ + ‘ to ‘x’). To quantify the level of change in odor representations over time in both states, we trained a classifier using data from time point 0 and tested it on data collected 4 weeks later, and vice versa (Figure 7C). The ability of a classifier to correctly identify an odor from neural activity recorded 4 weeks apart, decreased from 56 to 37% in the anesthetized state and from 52 to 33% in the awake state. In both cases classification accuracy remained much higher than chance level (Figure 7C). Importantly, these accuracies were both significantly higher than the accuracy across states, even for classification that was trained and tested on data collected in the same day (Figure 7C). These data suggest that, as compared to the changes during state transitions, odor representations remained relatively stable over time.

**FIGURE 8:**
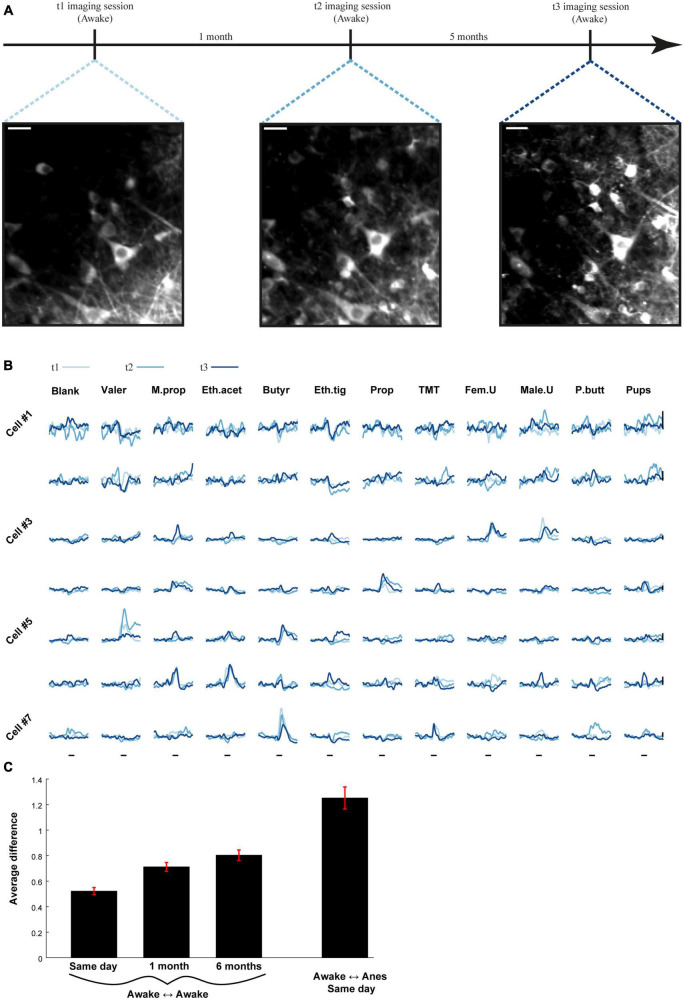
Odor representations are stable over long periods of time. **(A)** Time-line for the experiment used to assess stability of odor representations in one example mouse over 6 months. Two-photon micrographs of mitral cell (MC) imaged 6 months apart. Scale bar = 25 um. **(B)** Examples of odor-evoked calcium transients from seven MCs at three time points, spaced up to 6 months apart. Traces are averaged over five repetitions. Scale bar- vertical black line, 30% dF/F. Odor stimulus is denoted by a black line – 2 s. **(C)** The average difference between all cell-odor pairs recorded on the same day, 1 month apart and 6 months apart (three left bars). The difference between different states recorded on the same day (right bar). Awake to anesthetized recording was conducted between imaging sessions.

Finally, in one mouse, we examined the long-term stability of odor coding by MCs along three time points during wakefulness – 0, 4W and 24W (Figures 8A, B, 15 MCs from two fields). We quantified the distance between the traces of the same cell-odor pairs recorded in two sessions, using an average Euclidian distance, calculated over a 4 s response-window. The general similarity for the whole session was then calculated by averaging the distances between all cell-odor pairs (see section “Materials and methods”; smaller values represent higher similarity). This value of similarity suggests only a small representational drift between time points (Figure 8C). Notably, this representational drift is minor compared to the changes between states (Supplementary Figure 5). Although anecdotal, this experiment suggests that during a 6 months period, which is a significant portion of the life span of adult mice, the representational drift of odors by MCs is rather subtle.

## Discussion

We imaged odor-evoked responses of MCs during wakefulness, anesthesia, and across time. We found extensive changes in odor representations following the transition between these two states whereas within state, odor representation remained rather stable over time.

### Differences in odor representations across states

Some previous studies described the transition between anesthesia to wakefulness as one that induces sparser and more informative odor representations (Rinberg et al., 2006; Kato et al., 2012; Bolding et al., 2020), but some did not (Gheusi et al., 2000; Davison and Katz, 2007; Kollo et al., 2014; Economo et al., 2016). Unlike Kato et al. (2012), which found increased discriminability between odors in wakefulness (see Figure 2 in Kato et al., 2012), we found equal or slightly better odor discriminability during anesthesia (Figure 6). This difference may stem from the use of a more sensitive calcium indicator (GCaMP6) used here as compared to the previous study (GCaMP3). Specifically, once we consider both suppressed and excited responses, which can be readily measured using GCaMP6 but not GCaMP3, the total responsiveness in both states was similar (Figure 3A and Supplementary Figure 3D). Despite the general similarity in activity, odor representations across states were quite different. The difference across states was mainly expressed as a distinct normalization process across both stimuli and cells in the awake state, as well as a more balanced division of labor between E and S responses (Figure 3 and Supplementary Figure 3). Our imaging data is inconsistent with a recent electrophysiological study which found lower responsivity and a highly degraded odor code in the OB under anesthesia (Bolding et al., 2020). It is more difficult to reconcile the discrepancies when measurement methodologies are so different. We realize that calcium imaging provides a degraded proxy for general firing rates, which remains a general challenge in the field (Stringer and Pachitariu, 2019). As calcium sensors continue to improve, and imaging methods are ever more popular for assessing the functional properties of neuronal populations, it becomes necessary to improve the theory and experiments for inferring spiking from calcium activity (Rupprecht et al., 2021). This shortcoming is particularly evident in the absence of simultaneous imaging and electrophysiology from MCs in the OB.

As reported by others, our data also supports the notion that wakefulness is generally accompanied by increased levels of inhibition (Figure 3 and Supplementary Figures 2, 3; Rinberg et al., 2006; Kato et al., 2012; Cazakoff et al., 2014; Vinograd et al., 2017b; Wallace et al., 2017; Kudryavitskaya et al., 2021). However, we show that changes across states are highly odor-dependent. Indeed, the occasional odor (e.g., pup odors in our dataset) can consistently show stronger responses in the awake state as compared to anesthesia (Figures 4C, 5A). The general bias toward higher response magnitudes under anesthesia may also reflect the choice of odors in the dataset. Most studies in olfaction, and particularly those imaging the dorsal OB, use similar families of odors. In our dataset, we also chose six artificial odors that evoke strong odor response in the dorsal OB, and additional 5 “natural” odors some of which odors that mice may actually encounter. Additionally, we and others recently showed that natural odors show qualitatively distinct response profiles and plastic changes as compared to synthetic odors (Gschwend et al., 2016; Vinograd et al., 2017b). In this work, too, we observed some qualitative differences between how natural and synthetic odors change across states (Figures 5E, F).

### Awake or anesthetized?

While systems neuroscience is moving in a direction of recording in awake and behaving mice, animal preparations of anesthesia still have their merits. Studies interested in isolating brain signaling related to consciousness often use anesthesia as a model (Alkire et al., 2008; Bradley et al., 2022). In addition, anesthesia is useful if one is interested in measuring feedforward information while concurrently reducing activity from non-sensory sources (e.g., activity due to motion, attention, motivation, etc.). Indeed, measuring brain signals under anesthesia have been useful to reveal basic properties of single neurons in sensory areas, like neuronal tuning curves, which serve as basic building blocks of neural coding (Hubel and Wiesel, 1959; Davison and Katz, 2007). The regional differences of sensitivities to anesthesia can also carry information in a comparative context (Bolding et al., 2020). Nevertheless, since sensory representations are substantially different between wakefulness and anesthesia, projecting findings regarding basic sensory representations from one state to another, should be done with caution. Furthermore, substantial differences in behavior likely contribute to the difference in coding schemes between the states (e.g., body movement, sniffing patterns, etc.). Since we did not disentangle these factors from the observed neural activity, the nature of the comparisons between wakefulness and anesthesia remain inherently limited.

The finding that the transitions between wakefulness and anesthesia cause large changes in the very basic properties of single neurons is not surprising and has been discussed decades ago in olfaction and other sensory systems (Gaese and Ostwald, 2001; Fontanini and Katz, 2008; Bolding et al., 2020). In the visual thalamus of the mouse, receptive field properties of single neurons remained unchanged across states, but numerous attributes of their basic tuning properties did (Durand et al., 2016). Given that we still do not have a good understanding of the concept of odor space in olfaction, direct comparisons to other senses remains limited to tuning properties. Mapping odor space more exhaustively and with a wide range of concentration as has been done recently for olfactory sensory neurons will shed light on the nature of representations in the OB (Burton et al., 2022).

### Odor representations in the OB remain stable over time

The brain must maintain a balance between stability and plasticity in order to reliably perceive and act in a changing environment. While time lapse two photon imaging has limited temporal resolution it is particularly well suited to measure how neuronal structure and function change over time (Holtmaat and Svoboda, 2009). Here, we used two photon imaging to investigate the dynamics of representations of odors over long periods of time (6 months cover a significant portion of a lab’s mouse lifespan). Previous work show that distinct brain regions show different levels of dynamics in how they represent sensory information. On one hand, some brain regions seem to have highly stable representations by single neurons and populations (Dhawale et al., 2017; Katlowitz et al., 2018; Jensen et al., 2022; Liberti et al., 2022). On the other hand, other brain regions have been reported (often in contradiction to other reports) to show representational drift, such that population activity is reliable but single neurons show extensive representations drift (Lütcke et al., 2013; Clopath et al., 2017; Driscoll et al., 2017; Deitch et al., 2021). We find a relatively stable response of single neurons to odors in either awake or anesthetized states and a relatively stable representation of odors by the MC population as a whole (Figures 6–8; Bhalla and Bower, 1997).

Early studies by us and others used time lapse imaging of the OB to show that it is a brain region with a wealth of structural and functional changes (Adam and Mizrahi, 2010, 2011). In particular, the phenomenon of ongoing adult neurogenesis, forms a rich substrate for synaptic turnover (Whitman and Greer, 2009; Livneh and Mizrahi, 2011), including clear evidence for daily changes in MC synapses (Mizrahi, 2007; Livneh et al., 2009; Livneh and Mizrahi, 2012; Sailor et al., 2016). In this context, it may seem somewhat surprising that MCs maintain such high degree of functional stability. On the other hand, the precision with which axons of functionally similar olfactory sensory neurons project to single glomeruli is expected to keep feedforward inputs highly stable. Either way, the representational stability that we report for naïve mice may serve as solid reference for future plasticity by MCs. Indeed, there are myriad of examples for functional plasticity in the OB, that are invoked by a range of events, from associative learning to natural experiences (Doucette and Restrepo, 2008; Chu et al., 2016; Vinograd et al., 2017b; Yamada et al., 2017; Wu et al., 2020; Kudryavitskaya et al., 2021). Stable representations under baseline conditions reduces ongoing noise, and thereby magnifies the level of change when plasticity occurs. Thus, the extent of stability so early in the hierarchy may be valuable feature of the OB as an upstream region to steer downstream plasticity.

The relative stability of MCs does not seem to propagate downstream, at least not to the pirifom cortex under baseline conditions. Recent work showed that neurons in the piriform cortex, which is a major downstream target of MCs, exhibit robust representational drift. The performance of a classifier trained on data from the first day of recording, reached chance levels when tested on the 32nd day (Schoonover et al., 2021). Since over this time period MCs tuning is expected to be similar, the mechanism of ongoing plastic change in piriform cortex is likely found elsewhere. Both mechanisms, the one that continuously reshapes the neural code in piriform cortex despite a stable OB output, as well as the mechanisms that maintain MC’s in tune, remain to be explored by future experiments.

## Materials and methods

### Animals

All experimental procedures were approved by The Hebrew University Animal Care and Use Committee. Mice were kept on 12/12 light–darkness cycle. The strains we used were either wild type C57BL/6 mice or Nestin-Cre*^ERT^*^2^ [Jax stock #016261 (Lagace et al., 2007)] background strain C57BL/6 crossed to TB mice [Jax stock #031776, background strain FVB (Tasaka et al., 2018)]. Nestin-Cre*^ERT^*^2^ mice were not manipulated and since they are viable, fertile and normal in size they were treated as wild-types. Both females and males were used in the current research.

### Surgical procedures

We anesthetized mice with an intraperitoneal injection of ketamine and medetomidine (100 and 0.83 mg per kg, respectively) and a subcutaneous injection of carprofen (4 mg per kg). In addition, we injected mice subcutaneously with saline to prevent dehydration. We assessed the depth of anesthesia by monitoring the pinch-withdrawal reflex and added ketamine/medetomidine to maintain it when needed. We continuously monitored the animal’s rectal temperature at 36.5 ± 0.5°C. For calcium imaging, we made a small incision in the animal’s skin and glued a custom-made metal bar to the skull using dental cement to fix the head for imaging under the microscope. For acute imaging, we performed a craniotomy (2.5-mm diameter) over one OB. We poured 1.5% low-melting agar (type IIIa, Sigma-Aldrich) over the exposed brain, placed a glass cover over the craniotomy, and then secured it with dental cement. For awake experiments, we used triple layered window, following the procedure described others (Goldey et al., 2014).

### Virus injections

To express the Calcium indicator GCaMP6f in MCs, we used the following virus from Addgene - AAV5.CamKII.GCaMP6f.WPRE. SV40 (Cat# 100834-AAV5, 4.1 × 10^13^ genomic copies per ml). We injected virus directly into the left OB targeting the MCL (two injection sites per each bulb, ∼150 nl per site, at 200–500 μm depth) using Nanoject (Drummond Scientific).

### Odor delivery

For the odor stimulus presentation, we used a nine-odor air dilution olfactometer (RP Metrix Scalable Olfactometer Module LASOM 2), as described by others (Smear et al., 2013). Briefly, the odorants were diluted in mineral oil to 100 ppm. Saturated vapor was obtained by flowing nitrogen gas at flow rates of 100ml/min through the vial with the liquid odorant. The odor streams were mixed with clean air and adjusted to a constant final flow rate of 900ml/min. Odors were further diluted tenfold before reaching at a final concentration of 10 ppm to the final valve (*via* a four-way Teflon valve, NResearch). In between stimuli, 1000ml/min of a steady stream of filtered air flowed to the odor port continuously. During stimulus delivery, a final valve switched the odor flow to the odor port, and diverted the clean airflow to an exhaust line. Odors were delivered to the mouse nostrils *via* a custom-made glass mask, at a flow rate of 1 L/min (duration—2 s; interstimulus interval—26 s). Odors were continuously removed by air suction. The olfactometer was calibrated using a miniPID (Aurora Scientific).

For pure, non-natural odors we used a panel of six odorants known to activate different and partially overlapping areas in the dorsal part of the OB (Valeraldehyde [Pentanal], Methyl propionate, Ethyl acetate, Butyraldehyde [Butanal], Ethyl tiglate and Propanal; all obtained from Sigma-Aldrich, St. Louis, MO, USA). As five natural odorants we used: trimethylthiazoline (TMT), male urine, female urine, peanut butter, and nest odor. Urine was collected from both C57BL/6 and Nestin X TB males/females (∼5 different donors for each urine mixture) and stored at −20°C. Twenty-five microliter aliquots were placed in the odor vials and replaced between experiment on a daily basis. Peanut butter was made of 100% peanuts (Better and different, Mishor Edomim, Israel) and 1-g peanut butter was placed in the vials. For predator odor, we used 2 μl of TMT (Contech, Delta, Canada). Nest odor was made of 1-g nest bedding, collected from nests with new-born (∼2-5 days) pups. We stored pups bedding at −20° until the experiment, when we moved them into a vial in room temperature.

### Two-photon calcium imaging

We performed calcium imaging of the OB using an Ultima two-photon microscope from Prairie Technologies, equipped with a × 16 water-immersion objective lens (0.8 NA; CF175, Nikon). We delivered two-photon excitation at the 920nm using a DeepSee femtosecond laser (Spectraphysics). Acquisition rate was 7Hz. Before awake imaging and ∼3 weeks after implanting the window, we habituated the mice under the microscope in the head-fixed configuration.

In order to recover the same neurons on multiple sessions, we followed the same procedures that we recently described (e.g., Vinograd et al., 2017b; Shani-Narkiss et al., 2020; Kudryavitskaya et al., 2021). In short, in each mouse we found a clear anatomical mark on the surface of the OB (a blood vessel pattern), which was directly above the imaging field of view. This anatomical mark was used as an anchor and documented for future use. In a following session, the microscope was targeted to this anchor, and focusing down to the mitral cell layer revealed a region roughly including the previously imaged MCs. This region was compared to the previously stored micrograph of the field of view and manually aligned in the x, y, and z coordinates.

### Data analysis of the physiological experiments

We used Python 3.8 for the classifiers and population analysis (Figures 6, 7). In all other cases, we analyzed the data using Matlab (Mathworks). Movements were corrected using Moco plugin (Dubbs et al., 2016) (March 2016 release). Regions of interest corresponding to individual cell bodies were manually drawn and the mean fluorescence of each cell body was extracted by ImageJ at each frame and exported to Matlab for analysis. We calculated relative fluorescence change (*dF/F*), defining baseline fluorescence (*f*0) for each cell in each trial as its mean fluorescence measured 5–2s before odor onset. All traces were smoothed prior to analysis using Matlab’s default Smooth function, with a moving average filter with span = 5.

In order to determine the significance of response to an odor, a response window was defined as 0–4s post odor presentation, and local minima/maxima of the mean (over five repetitions) *dF*/*F* trace were detected, for cases in which the integral over the response window was negative/positive, respectively. The extremum point was taken together with six adjacent points, three from both sides. According to the type of extremum (minimum or maximum) picked at the response window, a parallel point from a baseline window ranging 6–2 s pre stimulus and a set of six adjacent points were chosen similarly. The effect size for each cell-odor pair was calculated as follows: $\frac{M\,e\,a\,n\,\left(r\,e\,s\,p\,o\,n\,s\,e\right)-M\,e\,a\,n\,\left(b\,a\,s\,e\,l\,i\,n\,e\right)}{m\,e\,a\,n\,\left(S\,t\,d\,\left(r\,e\,s\,p\,o\,n\,s\,e\right),S\,t\,d\,\left(b\,a\,s\,e\,l\,i\,n\,e\right)\right)},$ where “response” and “baseline” are vectors containing seven adjacent data points each, as described above. A response was classified as significant if it had effect size with absolute value bigger than five, and its magnitude was calculated as the integral over the five repetitions mean trace, 0–5 s post odor delivery. For the classification of response as suppressed or excited, the sign of the integral (±) determined the type of classification. In order to determine if a response was significantly different between states, a permutation test was conducted on two samples (one before and one after a change in state) for each cell-odor pair with at least one significant response (before and/or after), where each sample was composed of five numbers, representing the mean *dF/F* values averaged over the whole response window for each single trial. Comparisons resulting in *p* < 0.05 were counted as significantly different. Notably, multiple alternative classification methods conducted over the responses have all yielded qualitatively similar results to those reported at the final version of this work. Same is true for different definitions used for responses magnitudes (i.e., extremum vs. integral).

In order to compare suppressed and excited responses in experiments that were recorded in the same animal, and for further analysis conducted separately on these types of responses, responses were analyzed in either unpaired or paired manner, depending on context. When characterizing the networks at different states, we took unpaired responses (Figure 3 and Supplementary Figures 1, 2). Here, we included cell-odor pairs with significant response for each state separately, solely according to their response significance in that state. In other cases, we took responses in a paired manner, to better observe transformations of the same response in both states. In the paired analysis (Figures 4, 5 and Supplementary Figures 3, 4), every cell-odor pair classified with a significant suppressed/excited response was taken together with its before/after couple, regardless of its classification. To avoid mixing S and E responses in paired analyses, we omitted cell-odor pairs that exhibited significant S response in one condition and significant E response in the other condition. Thus, for plotting purposes only, we did not include responses that flipped direction between states. However, these instances constituted only ∼8% of the data set.

For calculating signal correlation, we calculated 11 scalar values for each cell (i.e., its responses to odors) by taking the integral over the mean dF/F trace during the response window. We then calculated pairwise correlations for all the cells recorded within the same mouse.

For the estimation of empirical tuning curves (Figures 5B–D), all cell-odor pairs were ranked and included, regardless their significance classification. Statistical tests were always two-sided, unless stated otherwise. Error bars always represent the standard error of the mean, unless stated otherwise. See Table 1 for complete details of all statistics.

**TABLE 1:** Details of statistical tests.

F	Test used	Mice (N)	Units(n)	*P*-value	d*f* and test statistic
3a	2-sample *t*-tests	Awake-11; Anes-20;	Awake– cells: 361; Anes.– cells: 700;	Total: 0.68; E resp: 3*e–5; S resp: 5*e–10;	Total: T_1059_ = 0.41; E resp: T_1059_ = –4.17; S resp: T_1059_ = 6.25;
3b	2-sample Kolmogorov-Smirnov test	Awake- 11; Anes- 20;	Awake- cells: 361; Anes.- cells: 700;	0.3	KS-2 stat = 0.063;
3c (#5)	2-sample t-tests followed by Bonferroni correction	Awake- 11; Anes- 20;	Awake-E resp: 86,88, 82, 90, 72, 65, 67, 79, 74, 95, 84; S resp: 64, 33, 38, 48, 91, 33, 58, 46, 48, 39, 49; Anes.– E resp: 134, 234, 146, 330, 501, 165, 175, 120, 144, 76, 105; S resp: 32, 78, 88, 49, 54, 61, 60, 35, 52,46, 41;	E resp: 0.08, 0.006, 0.132, 0.12, 0.004, 0.73, 0.00007, 0.00006, 0.0013, 0.0004, 0.00001; S resp: 0.00002, 0.72, 0.052, 0.013, 0.39, 0.1, 0.0007, 0.33, 0.1, 0.58, 0.055;	E resp: T_218_ = –1.76, T_320_ = –2.75, T_226_ = –1.5, T_418_ = –1.55, T_571_ = –2.85, T_228_ = –0.35, T_240_ = 4.04, T_197_ = 4.07, T_216_ = 3.24, T_169_ = 3.6, T_187_ = 4.5; S resp: T_94_ = –4.43, T_109_ = 0.36, T_124_ = –1.97, T_95_ = –2.53, T_143_ = –0.85, T_92_ = –1.66, T_116_ = –3.48, T_79_ = –0.98, T_98_ = –1.63, T_83_ = –0.56, T_88_ = –1.94;
3d (#5)	two-sample *t*-tests	Awake– 11; Anes.– 20;	Awake– E resp: 882; S resp: 547; Anes.– E resp: 2130; S resp: 596;	E resp:0.094; S resp:2*e–9;	E resp: T_3010_ = –1.68; S resp: T_1141_ = –6;
3e (#4)	*one–way anova* tests; odor–type x dF/F	Awake– 11; Anes– 20;	Awake– cells: 361; Anes.– cells: 700;	Awake: 2*e–7; Anes: < < 1*e–25;	Awake: F_10,360_ = 5.63; Anes: F_10,699_ = 151.95;
3f (#2)	2–sample *t*–tests	Awake– 11; Anes– 20;	Awake– E resp: 882; S resp: 547; Anes.– E resp: 2130; S resp: 596;	E resp: 9*e–6; S resp: 5*e–14;	E resp: T_3010_ = 4.4; S resp: T_1141_ = –7.63;
4d (#5)	1–sample *t*–tests followed by Bonferroni correction	7 mice,	E resp: 88, 82, 86, 107, 147, 72, 82, 66, 72, 113, 84; S resp: 31, 16, 27, 40, 42, 26, 31, 21, 23, 26, 20; Responses are paired;	E resp: 0.08, 0.013, 0.72, 2*e–8, 6*e20, 0.03, 0.002, 0.001, 7*e–6, 0.1, 6*e–6; S resp: 4*e–7, 0.96, 0.002, 0.64, 0.94, 0.89, 0.002, 0.001, 0.02, 0.44, 0.46;	E resp: T_87_ = 1.75, T_81_ = –2.54, T_85_ = –0.36, T_106_ = –6.03, T_146_ = –10.63, T_71_ = –2.22, T_81_ = 3.18, T_65_ = 3.37, T_71_ = 4.83, T_112_ = –1.64, T_83_ = 4.8; S resp: T_30_ = –6.44, T_15_ = 0.05, T_26_ = –3.35, T_39_ = 0.47, T_41_ = 0.08, T_25_ = –0.13, T_30_ = –3.23, T_20_ = –3.82, T_22_ = –2.52, T_25_ = –0.77, T_19_ = –0.76;
5A (#5)	Spearman Correlations followed by Bonferroni	7 mice;	249 cells;	0.032, 0.14, 0.59, 0.18, 3*e–6, 2*e–4, 0.034, 7*e–8, 0.094, 0.25, 4*e–5, 0.28;	Correlation coefficients are depicted in the figure, same as S4a.
5B (#4)	1 sample *t*–tests	7 mice;	249 cells;	Low: 2*e–10; High: 4*e–5 Deltas: 4*e–3.	Low: T_248_ = –6.63; High: T_248_ = –4.17; Deltas: T_248_ = –2.89;
6c (Py)	1–sample *t*–tests followed by Bonferroni	7 mice, sessions are paired;	7 mice	Anes. vs. Awake: 0.37 Anes. vs. diff state: 7*e–5 Awake vs. diff state: 0.001	Anes. vs. Awake: T_6_ = 0.98 Anes. vs. diff states: T_6_ = 9.5 Awake vs. diff states: T_6_ = 5.4
7c (#5)	1–sample *t*–tests and 2–sample *t*–tests, all followed by Bonferroni correction.	8 paired sessions from 4 mice, 8 sessions vs. 7 mice.	8 paired sessions. 8 sessions vs. 7 mice	Anes. vs. Awake: 0.68 Anes. vs. diff state: 0.004 Awake vs. diff state: 0.001	Anes. vs. Awake: T_7_ = 0.43 Anes. vs. diff states: T_13_ = 3.4 Awake vs. diff states: T_13_ = 4.1
S3a (#5)	1–sample *t*–tests	7 mice, paired responses	E resp: 999; S resp: 303;	E resp: 9*e–8; S resp: 4.4*e–6;	E resp: T_998_ = –5.38; S resp: T_302_ = –4.68;
S3d (#2)	1–sample Kolmogorov–Smirnov test	7 mice	249 cells;	0.054	KS stat = 0.08;
S3d (#2)	(Inset) 2–sample *t*–tests	7 mice	249 cells;	Total: 0.22; E resp: 1*e–4; S resp: 4*e–5;	Total: T_248_ = –1.22; E resp: T_248_ = –3.9; S resp: T_248_ = 4.1;
S3e (#5)	1–sample *t*–tests	7 mice	E resp: 999; S resp: 303; Responses are paired	E resp: 2.9*e–5; S resp: 8*e–4;	E resp: T_998_ = 4.2; S resp: T_302_ = –3.38;

### Analysis of population responses and odor discriminability

The temporal calcium response of each cell for each trail was converted to a 4-dimentional feature vector. These four features were extracted by using the first four principal components of all calcium traces in the entire dataset. These features capture both the magnitude of the response and the temporal profile of the response. We note that our result also hold for more or less than four features. Nearly identical results were received when using 2–7 features. Notably, the dimensionality reduction of the training data was unsupervised and, thus, did not affect the decoding results, for which we used supervised methods (see below).

For same-day odor discriminability, we used leave-one-trial-out cross validation procedure to assess accuracy i.e., we trained a logistic regression classifier on all cells (four features for each cell) using all available trials except one, and estimated the classification performance on the remaining trial. We repeated this process for all trials and averaged the results. This results in a single number per mouse. We then show results by averaging this number across available mice in the bar plots in Figures 6C, 7C.

For assessing discriminability within states at different time points, we trained a logistic regression classifier on all available sessions in one state and assessed performance on all sessions of the other state, i.e., using two sessions per mouse for assessing both possible directions. For the tSNE plots, we plotted the 2-dimensional tSNE of the entire population response (four features for each cell) for each mouse separately.

For plotting classification accuracy as a function of number of cells (Figure 6B), we grouped all recorded cells from all mice, we randomly selected 1 trial to serve as test trial and the remaining trials as training. We then randomly drew N number of cells, trained a logistic regression classifier on training trials, and made a prediction on the test trials. For each number of cells, we repeated this process 40 times, each time selecting a different random group of cells and a different random trial from each cell. Finally, we averaged these results and repeated the process for different values of N for each state.

For calculating response similarity between two sessions (Figure 8), we represented the average trace of each cell-odor pair as a 28-dimensional vector, utilizing 4 s x 7 Hz, and measured the Euclidean distance between traces. We then averaged this result across all pairs of responses, resulting in one value of similarity per each pair of sessions that we compared.

### Statistics

All statistical-tests and related values are shown in Table 1.

## Data availability statement

The raw data supporting the conclusions of this article will be made available by the authors, without undue reservation.

## Ethics statement

The animal study was reviewed and approved by the Hebrew University’s Institutional Animal Care and Use Committee.

## Author contributions

HS-N and AM designed the experiments. HS-N conducted the experiments and analyzed the data. DB carried out the modeling and related data analysis. AM and IS provided funding. HS-N and AM wrote the manuscript with input by DB and IS. All authors contributed to the article and approved the submitted version.

## Supplementary material

Supplementary Figure 1 Unpaired datasets: significant responses and variability between trials. **(A)** Significant calcium responses only, recorded from all awake (top) and anesthetized (bottom) mice in the unpaired datasets (see section “Materials and methods”). **(B)** Trial to trial variability, averaged per cell across all odors. Cells in the anesthetized state exhibited lower degree of trial-to-trial variability.

Supplementary Figure 2 Unpaired datasets: supplemental side-by-side comparisons. **(A)** Significant responses only in the unpaired datasets (same cell-odor pairs as in Figures 3A, B) normalized individually to their peak (E responses) or their valley (S responses) and sorted according to their type (S vs. E: top vs. bottom) and time to reach extremum. Full odor names are depicted in Figure 1A. Dashed lines represents the same extremum values that are plotted in Figure 3F. **(B)** Proportion of all responses (E and S combined) in anesthetized and awake sessions. Total number of responses was equal between states, but less stable across odors in the anesthetized state. **(C)** Proportion of E (bottom *x*-axis, left *y*-axis) and S (top *x*-axis, right *y*-axis) responses in anesthetized and awake sessions. **(D)** Distribution of peak dF/F values in S and E responses recorded in anesthetized and awake sessions. Inset- Cumulative distribution of the peak response amplitude for E (solid line) and S (dashed line) responses. Anesthesia causes a rightward shift for both types of responses, and a more significant shift to S responses.

Supplementary Figure 3 The paired dataset shows similar effects as the unpaired dataset. **(A)** Traces of significant S/E responses averaged across all odors in awake (blue) and anesthetized (red) states. Responses were taken in a paired manner (see section “Materials and methods”). Traces are presented as mean response (thick lines) shadowed by the SEM calculated overall average responses of a specific type at each time point. **(B)** Mean dF/F values, averaged throughout response window over all cells included in the datasets of the paired experiment. Net response magnitude was again more stable across odors in awake mice. **(C)** Proportion of E (lower *x*-axis, left *y*-axis) and S (higher *x*-axis, right *y*-axis) responses in anesthetized and awake sessions (red and blue, respectively). **(D)** Cumulative distribution of the proportion of the same MCs responding to 0–11 odors, in wakefulness (blue) and anesthesia (red). MCs responsiveness profile was not different between awake and anesthetized mice (*p* = 0.054, One-sample Kolmogorov–Smirnov test). Inset- Bars summarizing the differences in number of responses per cell between awake and anesthetized mice. S responses were more abundant in the awake state and E responses were more abundant in the anesthetized state, while the number of total responses per cell did not differ between states (*n* = 249 cells. S responses: *p* = 5.5 e-05; E responses: *p* = 1 e-04; Total responses: *p* = 0.22; Paired *t*-tests). **(E)** Top- histograms depicting times to reach maximum value for excited responses in awake and anesthetized states (*n* = 999 paired responses). Responses in anesthetized state peaked significantly earlier (paired *t*-test, *p* = 2.9 e-05). Bottom- histograms depicting times to reach minimum value for suppressed responses in awake and anesthetized states (*n* = 303 paired responses). Responses in awake mice reached minimum value significantly earlier (unpaired *t*-test, *p* = 8 e-04). Blue and red asterisks denote the mean time to extremum for awake and anesthetized sessions, respectively. **(F)** Same as Supplementary Figure 2A, but for data recorded in the same mice before and after anesthesia.

Supplementary Figure 4 Differences in MCs responses between awake and anesthetized states differ between monomolecular and natural odors. **(A)** Same as Figure 5A, but the transition is depicted in the opposite direction. **(B)** The change in response magnitude following anesthesia (*y*-axis) as a function of response magnitude in wakefulness. All responses are colored according to odor identity, as in 5E, and responses that did not differ significantly (gray circles- 5E) are now smaller in size. **(C)** The change in response magnitude following the transition to wakefulness (*y*-axis) as a function of response magnitude under anesthesia. **(D)** Distribution of the transitions between states of the cell-odor pairs. We include only responses that were significant in at least one state.

Supplementary Figure 5 Mitral cells (MCs) from the long time-lapse experiment imaged in awake versus anesthesia. Odor-evoked averaged calcium transients from the same MCs depicted in Figure 8 in awake (blue) and anesthetized (red) states. Scale bar- vertical black line, 30% dF/F.
