# Supplementary figures and images for: Stability and flexibility of odor representations in the mouse olfactory bulb
Source: Front Neural Circuits. 2023 Apr 20;17:1157259. doi: 10.3389/fncir.2023.1157259 (PMC10157098; doi:10.3389/fncir.2023.1157259)

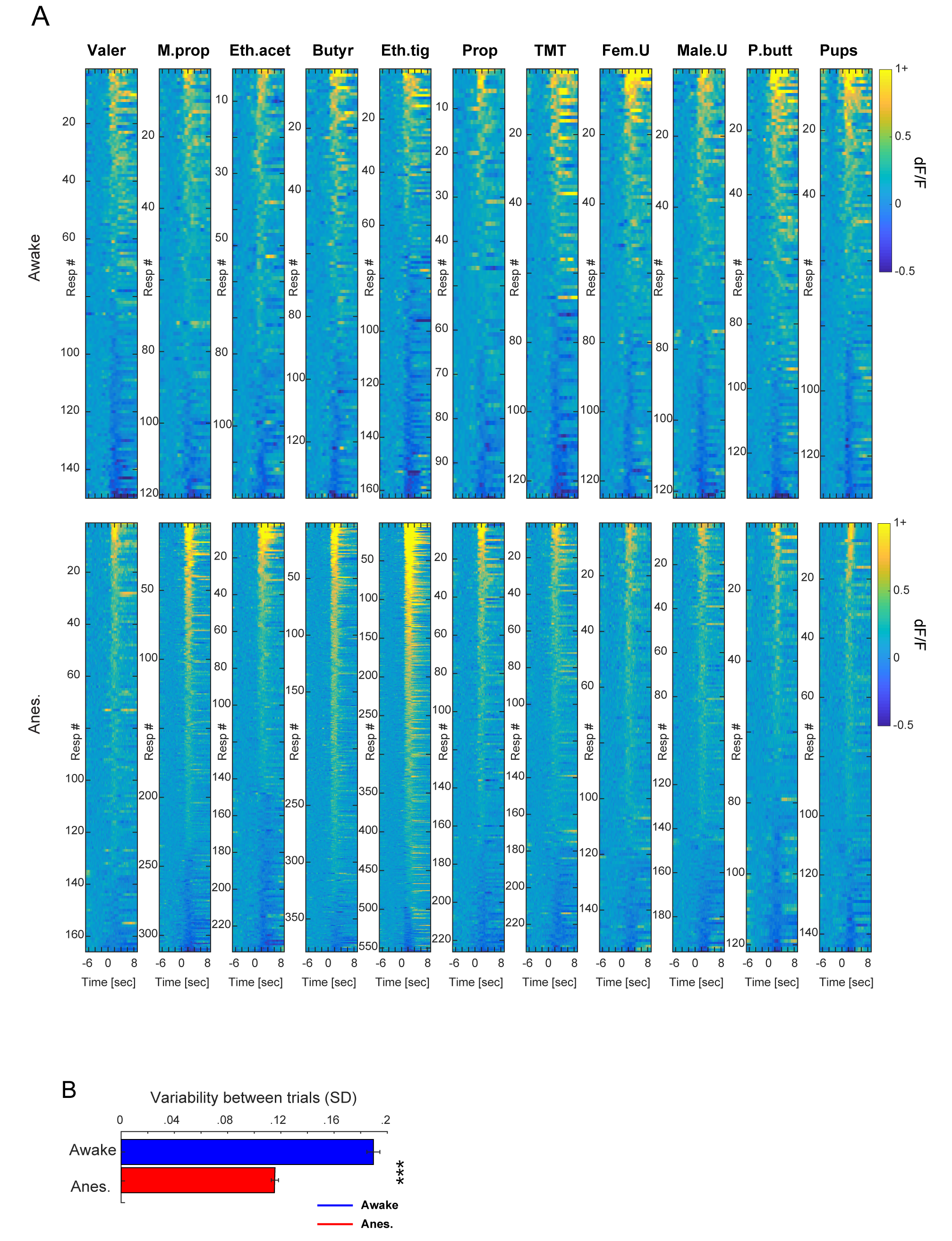

Supplement: Supplementary Figure 1 — Unpaired datasets: significant responses and variability between trials. (A) Significant calcium responses only, recorded from all awake (top) and anesthetized (bottom) mice in the unpaired datasets (see section “Materials and methods”). (B) Trial to trial variability, averaged per cell across all odors. Cells in the anesthetized state exhibited lower degree of trial-to-trial variability. [file Image_1.TIF]

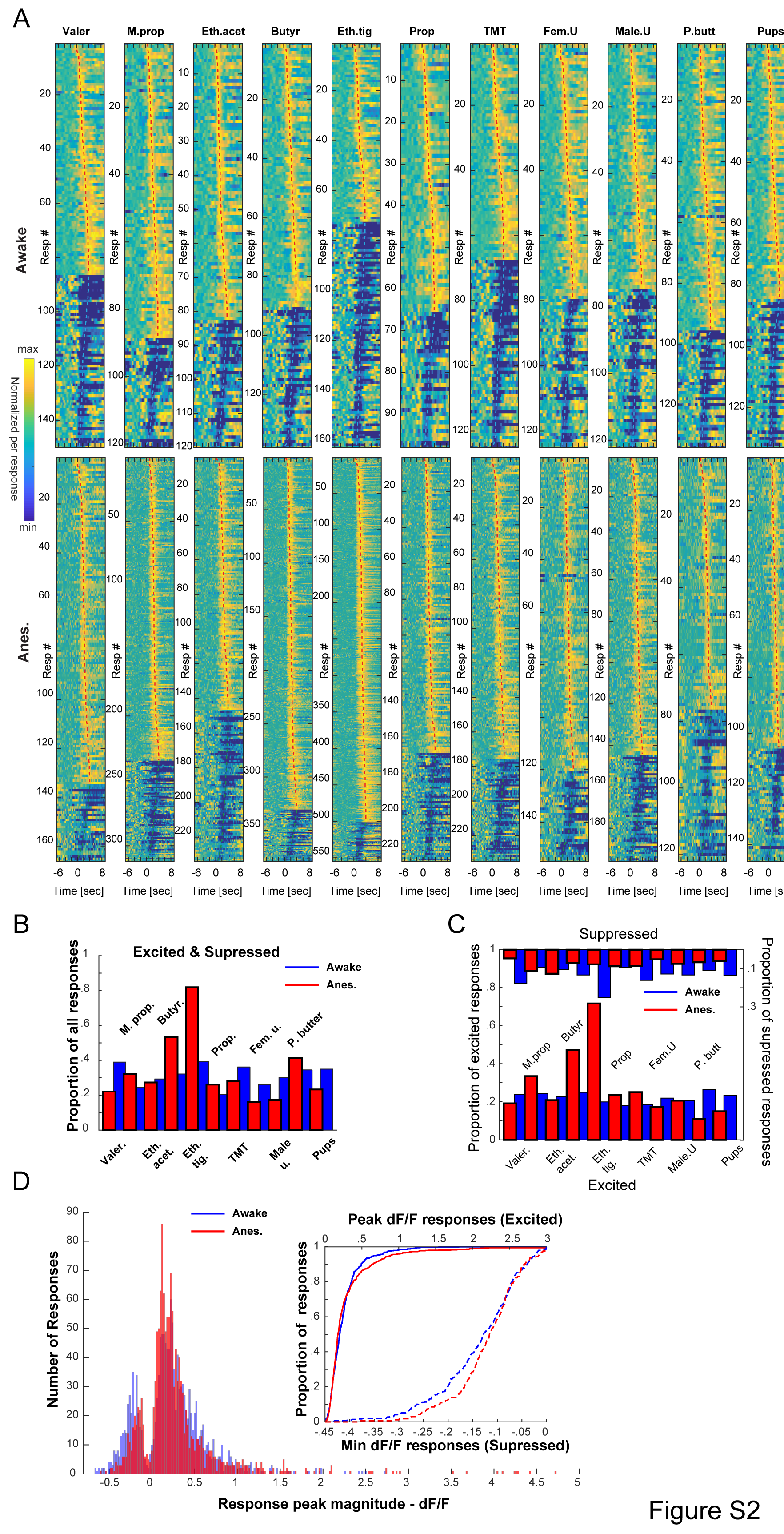

Supplement: Supplementary Figure 2 — Unpaired datasets: supplemental side-by-side comparisons. (A) Significant responses only in the unpaired datasets (same cell-odor pairs as in Figures 3A, B) normalized individually to their peak (E responses) or their valley (S responses) and sorted according to their type (S vs. E: top vs. bottom) and time to reach extremum. Full odor names are depicted in Figure 1A. Dashed lines represents the same extremum values that are plotted in Figure 3F. (B) Proportion of all responses (E and S combined) in anesthetized and awake sessions. Total number of responses was equal between states, but less stable across odors in the anesthetized state. (C) Proportion of E (bottom x-axis, left y-axis) and S (top x-axis, right y-axis) responses in anesthetized and awake sessions. (D) Distribution of peak dF/F values in S and E responses recorded in anesthetized and awake sessions. Inset- Cumulative distribution of the peak response amplitude for E (solid line) and S (dashed line) responses. Anesthesia causes a rightward shift for both types of responses, and a more significant shift to S responses. [file Image_2.TIF]

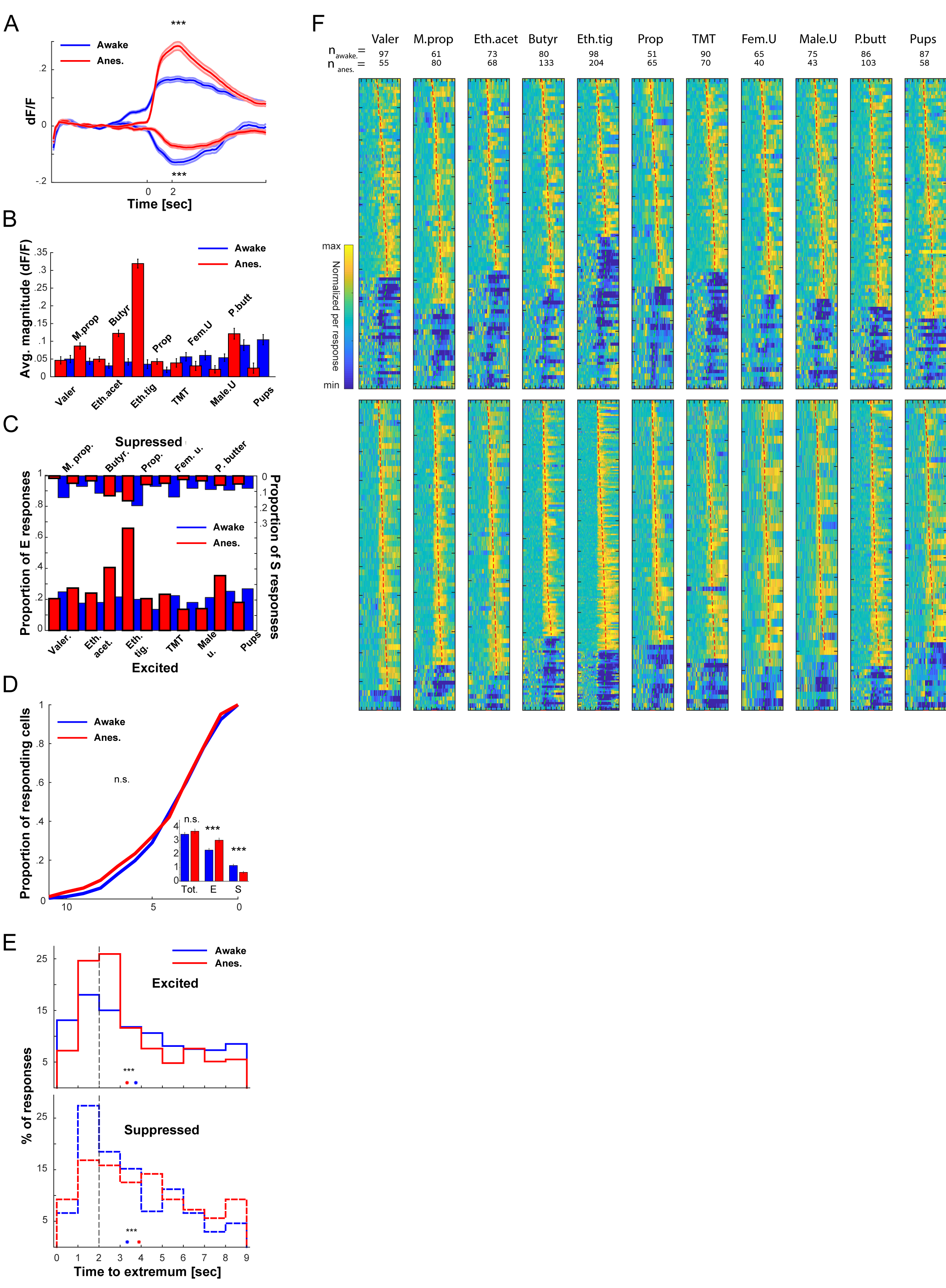

Supplement: Supplementary Figure 3 — The paired dataset shows similar effects as the unpaired dataset. (A) Traces of significant S/E responses averaged across all odors in awake (blue) and anesthetized (red) states. Responses were taken in a paired manner (see section “Materials and methods”). Traces are presented as mean response (thick lines) shadowed by the SEM calculated overall average responses of a specific type at each time point. (B) Mean dF/F values, averaged throughout response window over all cells included in the datasets of the paired experiment. Net response magnitude was again more stable across odors in awake mice. (C) Proportion of E (lower x-axis, left y-axis) and S (higher x-axis, right y-axis) responses in anesthetized and awake sessions (red and blue, respectively). (D) Cumulative distribution of the proportion of the same MCs responding to 0–11 odors, in wakefulness (blue) and anesthesia (red). MCs responsiveness profile was not different between awake and anesthetized mice (p = 0.054, One-sample Kolmogorov–Smirnov test). Inset- Bars summarizing the differences in number of responses per cell between awake and anesthetized mice. S responses were more abundant in the awake state and E responses were more abundant in the anesthetized state, while the number of total responses per cell did not differ between states (n = 249 cells. S responses: p = 5.5 e-05; E responses: p = 1 e-04; Total responses: p = 0.22; Paired t-tests). (E) Top- histograms depicting times to reach maximum value for excited responses in awake and anesthetized states (n = 999 paired responses). Responses in anesthetized state peaked significantly earlier (paired t-test, p = 2.9 e-05). Bottom- histograms depicting times to reach minimum value for suppressed responses in awake and anesthetized states (n = 303 paired responses). Responses in awake mice reached minimum value significantly earlier (unpaired t-test, p = 8 e-04). Blue and red asterisks denote the mean time to extremum for awake and anesthetized sessions [file Image_3.TIF]

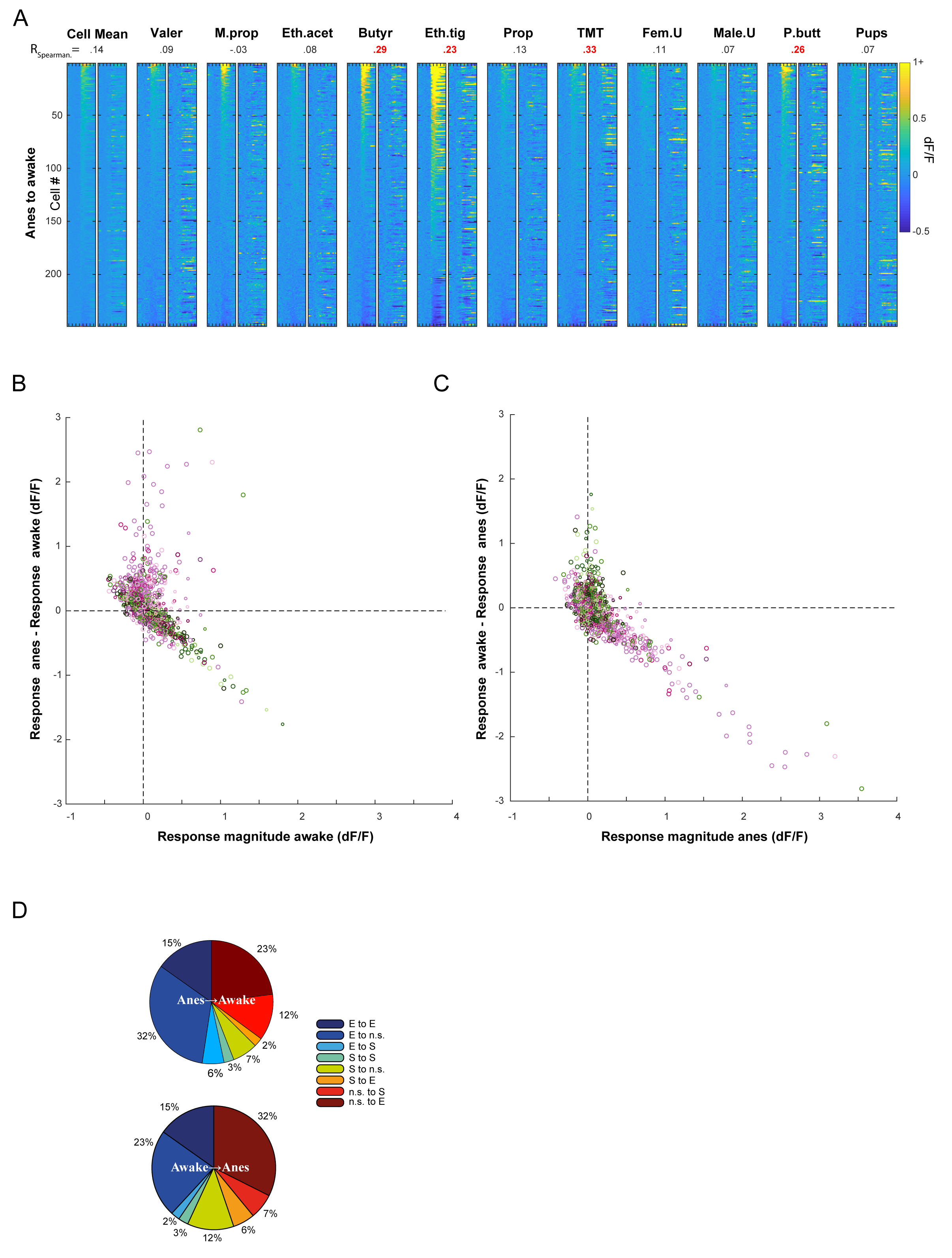

Supplement: Supplementary Figure 4 — Differences in MCs responses between awake and anesthetized states differ between monomolecular and natural odors. (A) Same as Figure 5A, but the transition is depicted in the opposite direction. (B) The change in response magnitude following anesthesia (y-axis) as a function of response magnitude in wakefulness. All responses are colored according to odor identity, as in 5E, and responses that did not differ significantly (gray circles- 5E) are now smaller in size. (C) The change in response magnitude following the transition to wakefulness (y-axis) as a function of response magnitude under anesthesia. (D) Distribution of the transitions between states of the cell-odor pairs. We include only responses that were significant in at least one state. [file Image_4.TIF]

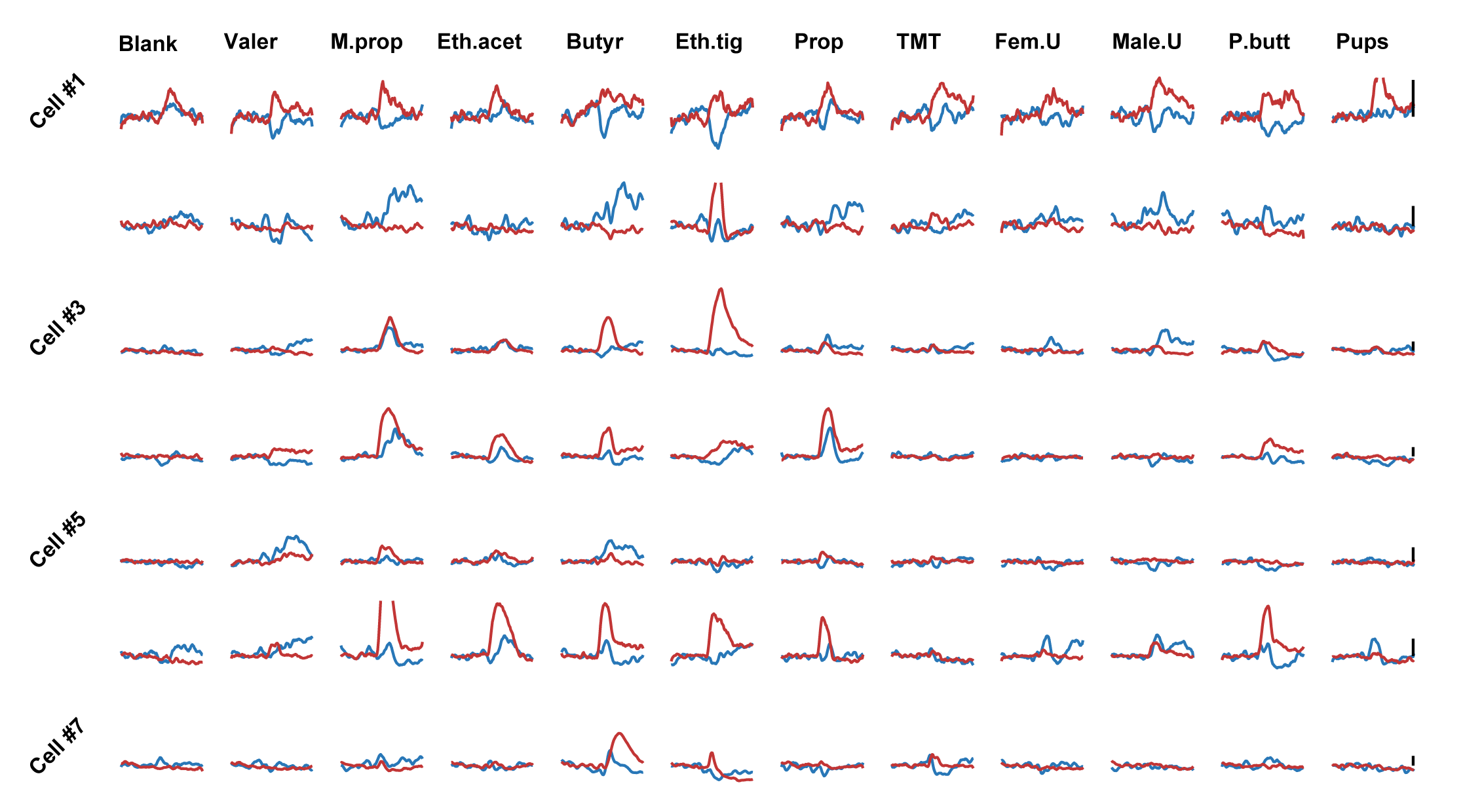

Supplement: Supplementary Figure 5 — Mitral cells (MCs) from the long time-lapse experiment imaged in awake versus anesthesia. Odor-evoked averaged calcium transients from the same MCs depicted in Figure 8 in awake (blue) and anesthetized (red) states. Scale bar- vertical black line, 30% dF/F. [file Image_5.tif]
